# The Long-Term Physical–Psychiatric Comorbidities Related to Childhood Exposure to 9/11 Trauma

**DOI:** 10.3390/ijerph21080988

**Published:** 2024-07-28

**Authors:** Lawrence Amsel, Yael M. Cycowicz, Diana V. Rodriguez-Moreno, Keely Cheslack-Postava, Larkin S. McReynolds, George J. Musa, Christina W. Hoven

**Affiliations:** 1 New York State Psychiatric Institute, New York, NY 10032, USA; yc60@cumc.columbia.edu (Y.M.C.);; 2 Department of Psychiatry, Columbia University, New York, NY 10032, USA; 3 Department of Epidemiology, Mailman School of Public Health, Columbia University, New York, NY 10032, USA

**Keywords:** comorbidity, trauma, children, 9/11, disaster

## Abstract

Extensive research has explored the enduring effects of childhood trauma on health, revealing its potential to produce chronic health problems. Despite findings that adults exposed to 9/11 suffer from enduring concurrent psychiatric and physical illnesses, investigations into the long-term physical–psychiatric comorbidities experienced by children and adolescents affected by the 9/11 trauma remain limited. In our study, we examined individuals directly exposed to 9/11 as children (N = 844 high exposure and N = 104 low exposed) and compared them to a matched unexposed, control group (N = 491). Fourteen years after their 9/11 exposure, we evaluated their physical and mental health conditions using parent- or youth self-reported health questionnaires, including psychiatric assessments. Those individuals with high 9/11 exposure were significantly more likely to have experienced a psychiatric disorder in the past year and a lifetime physical health condition compared to unexposed individuals. Moreover, the prevalence of physical–psychiatric comorbidities was higher among the 9/11-exposed group, with a 3.5-fold increased prevalence compared to the unexposed group. This underscores how exposure to traumatic events during childhood heightens the risk of long-term concurrent mental and physical health issues. Our findings also highlight the importance of early and ongoing interventions to prevent future comorbidities and promote better quality of life throughout the lifespan.

## 1. Introduction

Exposure to psychological traumatic events, especially those occurring during childhood, can exert profound long-term health effects, often leading to a complex interplay of physical conditions and psychiatric symptoms [1,2,3,4]. The persistent effects of psychological trauma can manifest as post-traumatic stress disorder (PTSD), depression, anxiety disorders, and other mood disturbances [5]. These conditions not only impact an individual’s day-to-day functioning but also contribute to a heightened risk of developing substance use disorders and self-destructive behaviors as coping mechanisms [6]. Critically, the consequences of psychological trauma are not confined to the realm of mental health, but also include a measurable impact on physical health, with trauma survivors frequently reporting a range of somatic symptoms, including headaches, gastrointestinal diseases, chronic pain, and sleep disturbances [7]. These and other physical ailments are partly the result of a chronic activation of the body’s multiple response systems, including the stress and immune systems. Chronically elevated stress hormones (e.g., cortisol) are known to contribute to an increased risk of cardiovascular problems and metabolic disorders [8]. In addition, trauma and its consequences can have long-term effects on the immune system [9]. As children experiencing trauma are susceptible to these long-term systemic effects [10], recognizing and addressing the multifaceted impact of childhood psychological trauma on well-being is crucial for designing effective interventions. Therefore, in this study, we have assessed the physical and psychiatric health of individuals that experienced the World Trade Center (WTC) terror attack as children, 14 years after their exposure.

The mass trauma of the WTC terrorist attack on 9 November 2001 holds unique significance in the American psyche, with significant political and social implications. It was the largest terrorist attack in US history, measured by the largest number of casualties and highly exposed survivors. From the perspective of long-term trauma research, the consequences of 9/11 continue to be assessed and hold a singular position by contributing significantly to our understanding of the short, medium, and long-term effects of mass trauma [11,12]. However, the vast majority of prior research focuses on the health impact among exposed adults [13,14,15,16,17], including, more recently, on their long-term physical–psychiatric comorbidities [18,19]. Across widely disparate exposure characteristics, exposed adults are increasingly more likely than unexposed peers to manifest an array of negative physical and psychiatric health outcomes, as well as their comorbidities [20,21,22,23]. In an examination of mortality among 9/11 first responders compared to adult civilians, Giesinger et al. [24] noted that 9/11-related PTSD appears to be associated with an increased risk of cardiovascular mortality, and proposed multiple mechanisms leading from PTSD to cardiovascular mortality. Similarly, Hamwey et al. [25] noted that, in addition to comorbid depression, the physical disorders of gastroesophageal reflux syndrome (GERS) and asthma were frequently comorbid with PTSD among adult 9/11 survivors. Many survivors of the 9/11 terror attack experienced dual exposures, to the attack as a psychological trauma and to the toxic air, which together were likely to contribute to the development of psycho-physical comorbidities [26]. One possible mechanism underlying these comorbidities involves inflammatory reprogramming triggered by both psychological trauma and toxicant exposure. This pathway can lead to physical health outcomes including sarcoidosis [27], obstructive airway disease [28], airway hyperreactivity [29], chronic rhinosinusitis [20], obstructive sleep apnea [30], prostate cancer [31], chronic kidney disease [32], gastroesophageal reflux symptoms, and co-occurring asthma [33], as well as other disorders that co-occur with the psychiatric outcomes of PTSD and mood disorders. However, even without toxicant exposure, trauma research suggests there is a causal link between psychological trauma and emergent somatic issues and comorbidities [34,35,36]. 

In contrast to the extensive body of literature on the effects of the WTC attack’s outcomes on adults, especially first responders, research on children and adolescents exposed to 9/11 is scarce [20,21,22,23]. A seminal study conducted by Hoven et al. [37], 6 months after the terror attack, reported that 28% of 8236 children in grades 4–12 in New York City public schools met the criteria for anxiety and depressive disorders. Other research on the health outcomes among exposed children and adolescents has primarily concentrated on either physical or mental health outcomes, with limited exploration of their comorbidity [4,38,39,40,41,42,43,44]. Retrospective studies found significant anxiety and depressive symptoms at 12 and 24 months after the terror attack, and an increased risk of suicidal ideation and PTSD even a year later in highly exposed adolescents [45,46]. Goodwin reported that the prevalence of probable panic attacks among highly exposed children was twice the prevalence among unexposed children [47]. Moreover, exposed adolescents who exhibited behavioral problems after the 9/11 attack endured long-term psychiatric symptoms suggesting probable PTSD, depression, or their comorbidity when they were assessed as adults [48]. Among younger children that were highly exposed to 9/11, the accumulation of trauma was associated with an increased emotional reactivity, symptoms of anxiety or depression, and sleep-related behavioral problems [49]. Additionally, exposure to the toxic dust produced by 9/11 among children living in lower Manhattan increased cases of asthma and worsened preexisting asthma symptoms [50,51,52]. Four years after the attack, young children’s exposure to the dust was associated with an increased rate of peripheral eosinophils, while adolescents experienced more new-onset GERD, headaches, and prehypertension [53]. While the majority of studies have focused on one type of health issues, a few emerging studies have reported on the comorbidities of psychiatric and physical symptoms [19,42,54]. Six to eight years after 9/11, over a third of adolescents with severe emotional distress and almost half of children with PTSD symptoms also had respiratory symptoms [54]. Similarly, 11 years after the attack, adults who were exposed as teens reported both an increased rate of mental disorders and respiratory problems [55]. Given the elevated rates of morbidity and mortality among those exposed to 9/11 in adulthood and the limited longitudinal research on children exposed to 9/11, it is reasonable to assume that such youths may be even more vulnerable to long-term mental and physical health comorbidity sequelae.

Danese and van Harmelen [56] noted that our understanding of the body’s response to childhood psychological trauma indicates it can trigger the same systemic physiological responses long known to be present in physical trauma. The definition of comorbidity within the public health and epidemiologic literature has been dynamically evolving, especially over the past twenty years. We now appreciate that, when examining the long-term effects of childhood trauma, it is critical to consider the duration of and overlap between the disorders involved relative to the developmental timeline. Many of the relevant disorders are chronic or have a life-long duration beginning in childhood, including, for example, asthma, epilepsy and diabetes. Similarly, many psychiatric disorders, like anxiety, depression, and eating disorders, have a chronic if not life-long course. Recent population-based studies found that there is a high risk for concurrent somatic and psychiatric illnesses in children of all ages and with diverse health disorders [57]. Similarly, a systematic review found a strong association between chronic somatic diseases and anxiety and depressive disorders in adolescents [58]. Research on adolescents has demonstrated a temporal chronology of the onset of mental disorders and physical diseases, where each can trigger the occurrence of the other [59,60]. Once initiated, the underlying physiological changes in the stress, metabolic, and immune systems associated with these conditions may persist, contributing to a coexistence of lifetime disorders. Therefore, the concept of comorbidity must encompass more than disorders with simultaneous onsets and persistence. Rather, they are often characterized by the occurrence of two chronic disorders over an extended period, not necessarily concurrent, as described by Valderas et al. [61]. Importantly, comorbidity has been linked to an increased burden of illnesses which impacts on quality of life and mortality [62]. Therefore, assessing the effects of childhood trauma over an extended period of time is crucial for understanding its potential impact on psychiatric and physical comorbidities. 

Consequently, the goal of this study was to examine the prevalence of comorbidity among a cohort of children/adolescents exposed to the 9/11 mass trauma compared to un-exposed youths. Our assessments included psychiatric disorders and symptom counts, as well as reports of physical symptoms and health-related complaints. We hypothesized that, fourteen years after 9/11, we would observe not only a higher prevalence of physical and psychiatric health issues, but also a higher rate of physical–psychiatric comorbidities, when comparing the highly exposed group to an unexposed control group. 

## 2. Methods

***Population.*** The Stress and Well-Being (S&W) cohort comprised N = 1000 individuals exposed to the 9/11 events as children (0 to 18 years of age on 11 September 2001), and N = 500 non-exposed community controls with similar demographic and family socioeconomic characteristics. The exposed sample was drawn as a representative sample from the World Trade Center Health Registry (WTCHR) Pediatric sample. The community controls were recruited from an area of New York City far removed from 9/11 Ground Zero, with similar demographic characteristics to the index sample. The Stress and Well Being (S&W) Study was conducted in the New York City Metropolitan Area, where all participants underwent assessment through a separate, confidential, in-home, face-to-face interview of the youth and their parent(s). The assessments were conducted, on average, 14 years following the 9/11 WTC attack. Data for the current analysis were obtained from 1439 participants with complete information for 9/11 exposure, and for physical and psychiatric health. The study was reviewed and approved by the New York State Psychiatric Institute IRB. Informed consent was obtained from parents for themselves and for their minor children, and children signed an assent form. Now-adult participants who were children at 9/11 signed their own consent form. 

***Demographic variables.*** Demographic information was based on youth reports and included biological sex, race/ethnicity (White, non-Hispanic; Black/African American, non-Hispanic; Asian non-Hispanic; Hispanic; and other races/multiple races/unknown), age, perceived household economic status (categorized into 3 levels: live very well; comfortably; or check to check/near poor).

***9/11 Exposure Status:*** Exposure to the 9/11 World Trade Center attack was classified into 3 categories based on reports from either children or parents (for children too young on 9/11 to recall). Subjects with “high” exposure were those who reported one or more of the following indicators: (a) having been enrolled in a school below Canal Street on 9/11; (b) having seen planes crash or the towers collapse with their own eyes; (c) having been in or near the cloud of smoke/dust; (d) having been physically hurt in the attack; or (e) having had to move out of their home as a result of the attack. Subjects not meeting “high” exposure criteria, were categorized as having “low/other” exposure. Subjects were labeled as “unexposed” if they were enrolled as community controls and reported none of the 9/11-related indicators.

***Assessment of physical and psychiatric health status:*** The interviews with parents and their children involved reports of physical diseases and assessments of psychiatric disorders. In most cases, psychiatric diagnoses relied on responses obtained from both parents and youths. However, if only one set of responses was available, whether from the parent or the child, diagnoses were based on the available information. 

*Assessment of Physical Diseases:* Parents and youth were given a list of physical diseases and they were asked to indicate if the child had any of them during the child’s lifetime. The physical disorders included meningitis or encephalitis, seizure or history of epilepsy, frequent ear infections, eczema, asthma, pneumonia, bronchitis, heart disease, diabetes, hypoglycemia, pernicious anemia, cancer, hepatitis, kidney disease, thyroid disease, HIV/AIDS, other STIs, strong stomach aches, ulcers, frequent headaches or migraines, or tuberculosis. 

*Assessment of psychiatric disorders*: The National Institute of Mental Health (NIMH) Diagnostic Interview Schedule for Children (DISC) [63] was used to assess past-year psychiatric diagnoses of generalized anxiety disorder, panic disorder, post-traumatic stress disorder (PTSD), agoraphobia, separation anxiety disorder, major depressive disorder, conduct disorder, oppositional defiant disorder, and substance use disorder (e.g., alcohol, marijuana, or other substance of abuse).

*Designation of Physical and Psychiatric Comorbidity Status*: For each participant, we defined a 4-level variable to represent health comorbidity status which includes (a) neither (no physical or psychiatric disorder), (b) only physical disorder, (c) only psychiatric disorder, (d) both physical and psychiatric disorder.

***Statistical analysis***: Study participant characteristics by 9/11 exposure group and by physical–psychiatric comorbidity status were compared using chi-squared tests or ANOVA for categorical and continuous variables, respectively. The association of 9/11 exposure level with the 4-level physical–psychiatric comorbidity status was then tested using multinomial logistic regression, with the “neither” category serving as the referent. Relative risk ratios (RRRs) and 95% confidence intervals (CIs) were estimated for each level of the comorbidity status associated with low or high 9/11 exposure, versus the unexposed group. Models were unadjusted and adjusted for covariates (biological sex, race/ethnicity, age, and perceived household economic status) to address potential confounding due to demographic characteristics. Wald tests were used to assess whether RRRs for the association of high 9/11 exposure with physical–psychiatric comorbidity differed statistically from RRRs for physical-only or psychiatric-only morbidity. Potential effect modification of associations by the participant age or biological sex was assessed by adding age X 9/11 exposure or biological sex X 9/11 exposure product terms to adjusted models. The children’s age at interview was highly correlated with their age at exposure, as the exposure occurred on one date and the interviews occurred within a limited time span. To test the sensitivity of the associations to psychiatric and physical morbidity based on both the parent and child reports, we repeated the adjusted analysis using a 4-level psychiatric–physical comorbidity variable based on youth report only. This analysis included data from subjects with complete information on this variable and all covariates. Analyses were conducted using SAS 9.4 (SAS Institute Inc., Cary, NC, USA).

## 3. Results

***Sample characteristics by 9/11 exposure status.*** The sample comprised 491 unexposed (control), 844 high 9/11 exposure, and 104 low 9/11 exposure subjects. The demographic characteristics of the sample by exposure status are shown in Table 1. The distributions of biological sex and perceived household economic status did not differ by 9/11 exposure status. However, the race/ethnic composition of the sample differed significantly by exposure group (*p* = 0.0004). White, non-Hispanic participants comprised the most prevalent category among the unexposed (40%) and the high 9/11 exposure (43%) group, whereas Hispanic participants were the most prevalent race/ethnic category among the low 9/11 exposure group. In addition, at the interview time, the exposure groups significantly differed by age (*p* < 0.0001), such that the low-exposure participants were younger than either the high exposure or unexposed groups. 

***Sample characteristics by comorbidity status.*** Table 2 shows the characteristics of the study population by physical–psychiatric comorbidity status. The physical–psychiatric comorbidity status groups differed significantly by race/ethnicity (*p* < 0.0001), household economic status (*p* = 0.02), and age at interview (*p* < 0.0001), but not by biological sex. 

***Overall Prevalence of Psychiatric and physical disorders by exposure status.*** The overall prevalence of past-year psychiatric disorders was 36% among highly exposed participants, which is significantly larger than the 28% among unexposed individuals (*p* = 0.004). Similarly, the overall prevalence of any lifetime physical health condition was 27% in the high exposure group, which is significantly larger than in the 10.6% in the unexposed group (*p* < 0.001).

**Figure 1:**
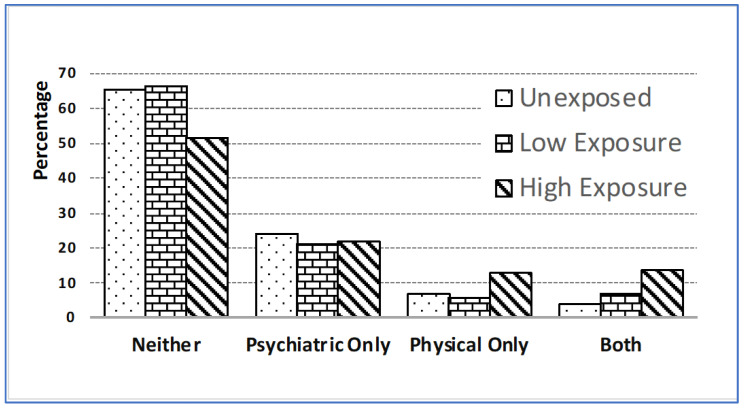
Physical–psychiatric comorbidity status by 9/11 exposure status.

***Prevalence of psychiatric-physical comorbidities by exposure status.*** Figure 1 presents the prevalence of physical–psychiatric comorbidities by exposure status. While the occurrence of only psychiatric disorders appears to be comparable across exposure groups, the combination of psychiatric and physical disorders is 3.5 times more prevalent in the highly exposed group than in the unexposed group (13.6% vs. 3.9%). This outcome highlights that the aforementioned differences in psychiatric morbidity can be attributed to the greater incidence of psychiatric–physical comorbidities. 

***Association of 9/11 exposure with physical–psychiatric comorbidity status***. The relative risk ratios (RRRs) for the association of 9/11 exposure level with psychiatric–physical comorbidity status are shown in Table 3. Compared to the unexposed group, high 9/11 exposure was associated with an over 4-fold increased relative risk of psychiatric–physical comorbidity (adjusted RRR = 4.60, 95% CI = 2.75, 7.71), and an over 2-fold increased relative risk of physical-only morbidity (adjusted RRR = 2.49, 95% CI = 1.63, 3.81). However, psychiatric-only morbidity was not significantly increased among the highly exposed compared to the unexposed (adjusted RRR = 1.21, 95% CI = 0.92, 1.60). Furthermore, among the high exposure group, the relative risk of psychiatric-physical comorbidity was significantly greater than the relative risk of physical morbidity only (*p* = 0.05) or of psychiatric morbidity only (*p* < 0.0001). The addition of either (a) participant biological sex × 9/11 exposure product terms or (b) participant age at 9/11 × 9/11 exposure product terms to the adjusted models shown in Table 3 to assess evidence for effect modification by biological sex or age at 9/11 exposure did not suggest evidence for either (*p*-values for interaction, *p* = 0.63 for biological sex and *p* = 0.73 for age at 9/11 exposure). The unadjusted model yielded similar results. Sensitivity analyses using an outcome definition based only on youth report of psychiatric and physical health outcomes also showed that high 9/11 exposure was associated with a significantly elevated risk of psychiatric–physical comorbidity (adjusted RRR = 1.62, 95% CI = 1.08, 2.41), and this differed significantly from the relative risk of physical-only outcomes (*p* = 0.04).

## 4. Discussion 

The results of this study support the hypothesis that exposure to mass trauma during childhood and adolescence may heighten the risk of developing multiple health conditions. While we observed an overall greater prevalence of psychiatric and physical issues in the highly exposed individuals compared to the unexposed ones, it is the notable disproportion of 3.5 times higher prevalence of psychiatric–physical comorbidities among the highly exposed group that underscores the extensiveness of the health ramifications of trauma exposure. Furthermore, these findings are consistent with the hypothesis that traumatic exposures during development may induce systemic alterations in the stress, endocrine, and immune systems contributing to this adverse health trajectory [64]. 

There has been a growing interest in unraveling the complexities of psychiatric–physical comorbidity. Studies in adults have extensively documented the simultaneous occurrence of physical ailments and mental disorders [7,34,35]. Historically, these lifelong disruptions, originally conceptualized by Hans Selye as the “General Adaptation Syndrome,” have been postulated to be induced by various stressors and to pose broad physical and mental health risks later in life [65]. A meta-analysis confirmed the presence of long-lasting inflammatory changes in chronic somatic and neuropsychiatric disorders within trauma-exposed groups [66]. Studies among adult survivors and first responders of the 9/11 attacks have also shown a long-term development of both physical and psychological sequelae, as well as mind–body comorbidities [19]. In their review of the WTC literature on psychiatric sequelae, Hamwey and colleagues [25] highlighted that many studies suggest that 9/11-exposed individuals are facing comorbid conditions, yet there is inadequate exploration of the interplay between the two. Among those that have investigated comorbidities in childhood trauma, Danese and van Harmelen [56] emphasized the unique role that PTSD plays in the realm of physical–psychiatric comorbidity, supporting the idea that a mental illness resulting from psychological trauma, such as PTSD, can precipitate subsequent physiological dysfunctions. The increased prevalence of comorbidities in our sample aligns with these theoretical considerations as well as with the adult WTC literature. Although particular associations may exist between certain traumas and the temporal sequence of physical/psychiatric disorders, there is consensus that trauma leads to long-term changes in multiple biological pathways, including the stress response and inflammation systems. Holz et al. [67] highlighted that comorbid physical–psychiatric outcomes may indicate common, systemic, and persistent pathways that “remember” and longitudinally respond to trauma and adversity. Thus, trauma exposure such as 9/11 can serve as a potential source of both physical and psychological dysfunctions, increasing the risk of comorbidities. Moreover, early trauma may precipitate even more enduring physical and neurological changes, exacerbating the risk for comorbidities. 

Longitudinal studies suggest that adopting a temporal approach to comorbidities can aid in understanding whether specific physical disorders precede and cause psychiatric disorders, or vice versa. For instance, Tegethoff and colleagues [59] examined the chronology of the onset of mental disorders and physical diseases in mental–physical comorbidity using a national representative survey of adolescents (N = 6830). Contrary to the traditional perspective that comorbidities originate with physical disorders leading to mental health conditions, they concluded that mental disorders early in life also serve as risk factors for physical diseases. In our study, the observed a 3.5-fold increase in physical–psychiatric comorbidity among the high exposure group compared to the unexposed group surpassed our expectations, indicating the pivotal role of developmental timing in the emergence of physical–mental comorbidities. Compelling evidence, including meta-analyses [66,68], underscores the linkage between childhood adversity and adverse physical, mental, and behavioral health outcomes [69,70,71,72,73,74]. While Adverse Childhood Experiences (ACEs) often entail diverse exposure types over prolonged periods, differing from the acute trauma of 9/11, research on ACEs has consistently identified comorbidity as a common outcome, independently echoing our findings. The presence of long-term health effects of 9/11 affects both children and adults well beyond the mass trauma event, but it is plausible that the mechanisms and impact differ between these age groups. Early trauma may alter the overall adaptational stance of a developing child far more, signaling that it is inhabiting a challenging environment, and thus adjusting development accordingly. This results in both somatic and psychiatric changes which are intended to be adaptive, but which may also manifest as disorders.

The strengths of this study must be weighed against its limitations. We conducted this study with a relatively large sample of individuals exposed as children or adolescents to a single shared traumatic event. The shared mass trauma reduces the variability related to trauma exposure observed in studies focusing on distinct individual traumas. We employed extensive and rigorous psychiatric instruments for mental health assessment. Unlike many studies with traumatized populations, we included a control group, enabling a comparison between highly exposed and unexposed individuals rather than solely those with low exposure levels. However, our assessment of physical morbidities relied on self-reports from the adult and adolescent participants and their parents asked about symptoms or conditions but lacked clinical assessments or healthcare records. This introduces the potential for differences in recall of health symptoms by participants and their parents, potentially biasing our results. Addressing this possibility necessitates the use of objective measures for assessing both psychiatric and physical health. 

Another limitation is that physical disorders were assessed over a lifetime, leaving open the possibility that some disorders occurred before the 9/11 exposure. In contrast, psychiatric disorders were assessed within the past year rather than since the time of exposure, potentially leading to an underestimation of their prevalence. Thus, the lack of complete psychiatric and physical health status information over the life course of the child or adolescent precluded chronological analysis of these comorbidities. Nevertheless, since our results hinge on the difference between two comparable groups differing in their exposure levels, the misclassification of pre-exposure physical disorders and post-exposure psychiatric disorders are expected to be the same across these groups. Future prospective studies should incorporate pre- and post-exposure mental and physical assessments and obtain health records to further support our conclusions regarding comorbidity.

## 5. Conclusions

Our findings provide significant empirical support for emerging models of the long-lasting effects of trauma by focusing on a single shared traumatic exposure during childhood. We highlighted the pivotal role that trauma plays in shaping the developmental and long-term comorbidity patterns observed in these individuals. Contrary to the popular saying “that time heals all wounds”, time alone does not alleviate the impacts of childhood trauma. Therefore, it is imperative that individuals exposed to trauma during childhood or adolescence receive ongoing follow-up evaluations, preventative interventions, and treatment. Without such attention, they remain at lifelong risk of developing physical and psychiatric comorbidities and having a diminished quality of life.

## Figures and Tables

**Table 1:** Sample characteristics by 9/11 exposure status.

Characteristic		Unexposed N = 491	Low Exposed N = 104	High Exposed N = 844	*p*-Value
		N (%)	N (%)	N (%)	
Race/ethnicity	White, non-Hispanic	198 (40.3)	24 (23.1)	364 (43.1)	0.0004
	Black, non-Hispanic	26 (5.3)	9 (8.7)	80 (9.5)	
	Asian, non-Hispanic	68 (13.8)	22 (21.2)	118 (14.0)	
	Hispanic	138 (28.1)	38 (36.5)	203 (24.1)	
	Other/multiple races/unknown	61 (12.4)	11 (10.6)	79 (9.4)	
Biological Sex	Male	247 (50.3)	49 (47.1)	410 (48.6)	0.76
	Female	244 (49.7)	55 (52.9)	434 (51.4)	
Perceived household economic status ^a^	Live very well	84 (17.1)	18 (17.5)	183 (21.8)	0.28
	Live comfortably	320 (65.2)	68 (66.0)	525 (62.6)	
	Check to check/near poor	87 (17.7)	17 (16.5)	131 (15.6)	
Age at interview [mean (sd)]		21.5 (5.0)	19.1 (5.5)	22.4 (5.5)	<0.0001

^a^ Missing data for n = 6 observations.

**Table 2:** Sample characteristics by physical–psychiatric comorbidity status.

Covariate		Neither N = 825	Psychiatric Only N = 325	Physical Only N = 148	Both N = 141	*p*-Value
		N (%)	N (%)	N (%)	N (%)	
Race/ethnicity	White, non-Hispanic	321 (38.9)	140 (43.1)	70 (47.3)	55 (39.0)	<0.0001
	Black, non-Hispanic	65 (7.9)	19 (5.8)	11 (7.4)	20 (14.2)	
	Asian, non-Hispanic	161 (19.5)	34 (10.5)	9 (6.1)	4 (2.8)	
	Hispanic	188 (22.8)	92 (28.3)	47 (31.8)	52 (36.9)	
	Other/Multiple races/unknown	90 (10.9)	40 (12.3)	11 (7.4)	10 (7.1)	
Biological Sex	Male	416 (50.4)	152 (46.8)	77 (52.0)	61 (43.3)	0.29
	Female	409 (49.6)	173 (53.2)	71 (48.0)	80 (56.7)	
Perceived household economic status ^a^	Live very well	177 (21.6)	67 (20.6)	20 (13.7)	31 (14.9)	0.02
	Live comfortably	530 (64.6)	198 (60.9)	95 (65.1)	90 (63.8)	
	Check to check/near poor	114 (13.9)	60 (18.5)	31 (21.2)	30 (21.3)	
Age at interview [mean (sd)]		21.3 (5.8)	21.9 (5.4)	23.5 (4.0)	23.2 (4.1)	<0.0001

^a^ Missing data for n = 6 observations.

**Table 3:** Association of psychiatric-physical comorbidity with 9/11 exposure.

	9/11 Exposure	Psychiatric Only	Physical Only	Both
		RRR * (95% CI)	RRR (95% CI)	RRR (95% CI)
Unadjusted model	Unexposed	1.0 (ref)	1.0 (ref)	1.0 (ref)
	Low	0.87 (0.51, 1.47)	0.85 (0.34, 2.10)	1.71 (0.69, 4.23)
	High	1.16 (0.88, 1.52)	**2.43 (1.61, 3.69)**	**4.46 (2.69, 7.40)**
Adjusted model **	Unexposed	1.0 (ref)	1.0 (ref)	1.0 (ref)
	Low	0.97 (0.57, 1.66)	1.07 (0.42, 2.69)	1.91 (0.76, 4.80)
	High	1.21 (0.92, 1.60)	**2.49 (1.63, 3.81)**	4.60 *** (2.75, 7.71)

* RRR, relative risk ratio vs. neither psychiatric no physical morbidity, estimated using multinomial logistic regression. ** Adjusted for gender, age, race/ethnicity, and household economic status. *** Differs from RRR for psychiatric only (*p* < 0.0001) and physical only (*p* = 0.05).

## Data Availability

The raw data that support the findings of this study will be available as per the data sharing agreement between NYSPI and CDC/NIOSH.
